# Taxonomic notes on the genus *Eupoa* Żabka, 1985 (Arachnida, Araneae, Salticidae)

**DOI:** 10.3897/zookeys.410.7548

**Published:** 2014-05-21

**Authors:** Dmitri V. Logunov, Yuri M. Marusik

**Affiliations:** 1 The Manchester Museum, The University of Manchester, Oxford Road, Manchester M13 9PL, UK; 2 Institute for Biological Problems of the North FEB RAS, Portovaya Str. 18, Magadan 68500, Russia; 3 Zoological Museum, University of Turku, FI-20014 Turku, Finland

**Keywords:** SE Asia, jumping spiders, Aranei, *Eupoa*, new species, (re)descriptions

## Abstract

The south-east Asian genus *Eupoa* is redescribed and diagnosed. Seven new species are diagnosed, described and illustrated: *E. daklak* **sp. n.** (♀) from Viet-Nam; *E. lehtineni* **sp. n.** (♂♀) from India, Thailand and Viet-Nam; *E. lobli* **sp. n.** (♂) from Malaysia; *E. pappi* **sp. n.** (♂) from Thailand; *E. pulchella* **sp. n.**(♂) from Thailand; *E. schwendingeri* **sp. n.** (♂♀) from Thailand; and *E. thailandica* **sp. n.** (♂♀) from Thailand. *Eupoa prima* Żabka, 1985 and *E. yunnanensis* Peng & Kim, 1997 are redescribed and illustrated on the basis of type and/or newly collected materials. The female of *E. yunnanensis* Peng & Kim, 1997 is found and described for the first time.

## Introduction

The genus *Eupoa* Żabka, 1985 was described to accommodate a single species *Eupoa prima* Żabka, 1985 from northern Viet-Nam (Żabka 1985). For more than 10 years the genus had been considered monotypic until three more species were described by Peng and Kim (1997) from China (Hainan and Yunnan provinces): *Eupoa liaoi* Peng & Li, 2006; *Eupoa maculata* Peng & Kim, 1997 and *Eupoa yunnanensis* Peng & Kim, 1997. Later, three more species were described from China: *Eupoa liaoi* Peng & Li, 2006 from Hainan province (Peng and Li 2006) and *Eupoa jingwei* Maddison & Zhang, 2007 and *Eupoa nezha* Maddison & Zhang, 2007 from Guangxi (Maddison et al. 2007). Two of the *Eupoa* species described from Hainan (*Eupoa liaoi* and *Eupoa maculata*) have recently been transferred to other genera (Zhou and Li 2013a). Thus, to date a total of five valid species has been described in the genus *Eupoa*: one from northern Viet-Nam and four from south-eastern regions of China (Yunnan, Guangxi and Hainan).

By their general appearance, members of the genus *Eupoa* are superficially similar to those of *Neon* Simon, 1876. Both groups are small to very small spiders, with males having the swollen and relatively large palps for the size of the spiders (Figs 42–43, 86, 90). Both *Eupoa* and *Neon* are typical dwellers of forest leaf litter. However, the conformation of male copulatory organs in *Eupoa* is much more complex leaving no doubts that both genera are not related. The phylogenetic relationships of *Eupoa* remain poorly resolved, although the genus is indeed a basal salticid; see below under ‘Diagnosis and Affinities’.

The aim of the present paper is twofold: (1) to provide a comprehensive description of the genus *Eupoa* on the basis on newly collected materials; and (2) to describe seven new species from various regions of SE Asia.

## Material and methods

A total of 42 adult museum specimens belonging to nine species has been examined. The material studied in this paper was borrowed from the following museums: HNHM = Hungarian Natural History Museum, Budapest, Hungary (curator: Dr T. Szuts); MHNG = Museum d’Historie Naturelle, Geneve, Switzerland (curator: Dr P. Schwendinger); SMFM = Naturmuseum und Forschungsinstitutt Senckenberg, Frankfurt am Main, Germany (curator: Dr P. Jäger and Ms J. Altmann); ZMTU = Zoological Museum, Turku University, Turku, Finland (curator: Mr S. Koponen); ZMUM = Zoological Museum of the Moscow State University, Moscow, Russia (curator: Dr K. G. Mikhailov).

Digital photographs were taken using an Olympus E-520 camera attached to an Olympus SZX16 stereomicroscope, and prepared using CombineZP image stacking software. Photographs were taken with the specimens secured in dishes with paraffin on bottom. SEM microphotographs were made by means of a JEOL JSM-5200 in the Zoological Museum, University of Turku. Epigynes were cleared in a 10% KOH solution.

Abbreviations used in the text and figures are as follows: *Eyes*: AME – anterior median eye, ALE – anterior lateral eye, PME – posterior median eye, PLE – posterior lateral eye. *Copulatory organs*: CTA – compound terminal apophysis; E – embolus; ID – insemination ducts; MA – median apophysis; PA – patellar apophysis; R – receptacle; TA – tegular apophysis; TbA – tibial apophysis. *Leg segments*: Fm – femur, Pt – patella, Tb – tibia, Mt – metatarsus, Tr – tarsus. *Position of leg spines*: d – dorsal, pr – prolateral, rt – retrolateral, v – ventral. For the leg spination the system adopted is that used by Ono (1988). The sequence of leg segments in measurement data is as follows: femur + patella + tibia + metatarsus + tarsus. All measurements are in mm.

## Taxonomy

### Genus Eupoa

Żabka, 1985

http://species-id.net/wiki/Eupoa

Eupoa Żabka, 1985: 220. Type species: *Eupoa prima* Żabka, 1985; by monotypy.

#### Description.

**Figures 1–8.**
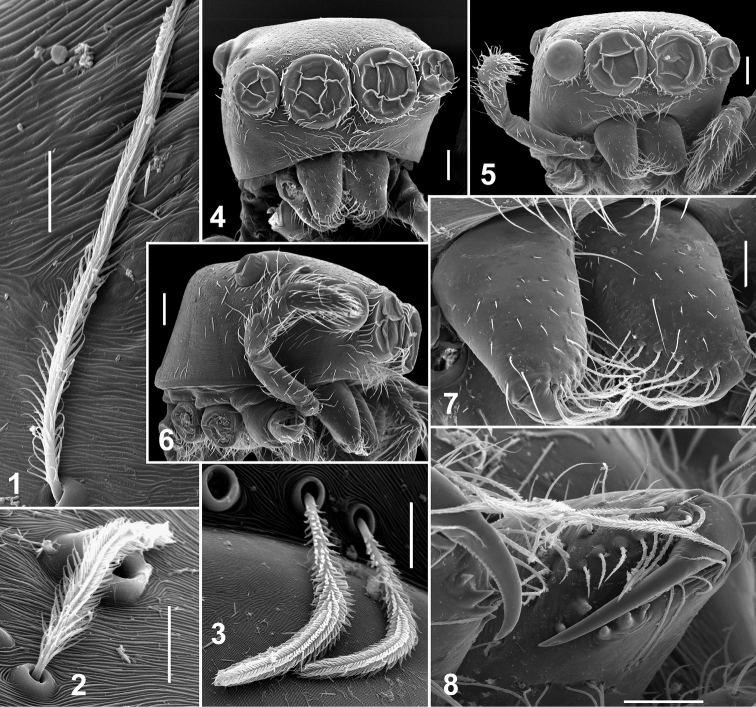
Somatic characters of *Eupoa lehtineni* sp. n. **1–3** plumose scales on female carapace. **4** male carapace, frontal view **5** female carapace, frontal view **6** ditto, lateral view **7** female chelicerae, frontal view **8** female fang and cheliceral teeth. Scale bars: 10 μm (**1–3**), 50 μm (**7–8**), 0.1 mm (**4–6**).

**Figures 9–16.**
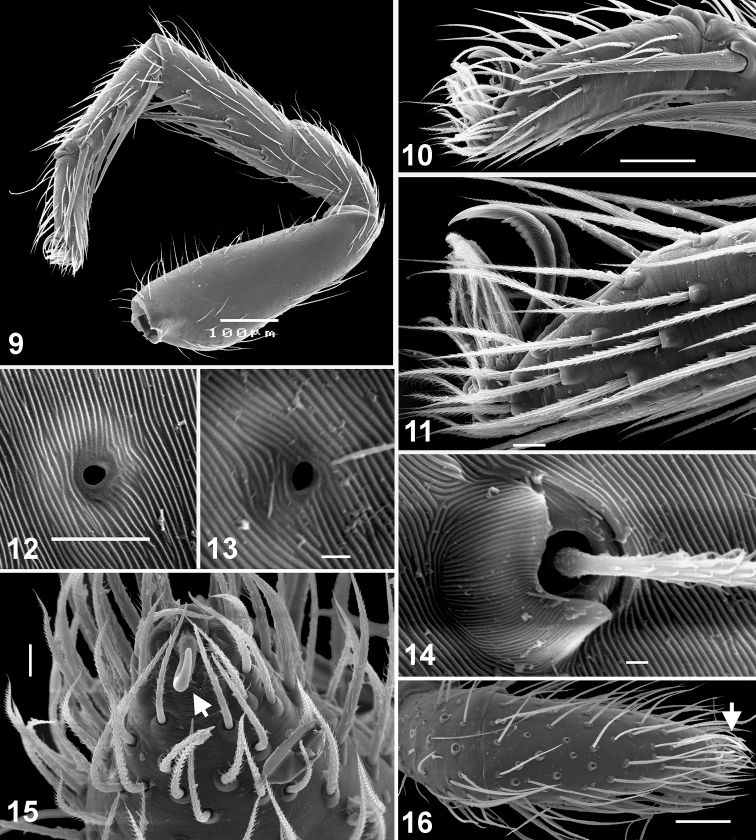
Somatic characters of *Eupoa lehtineni* sp. n. (**9–11, 13–16**) and *Eupoa thailandica* sp. n. (**12**). **9** female leg I, median view **10** female tarsus I, lateral view **11** female tarsus III, lateral view **12–13** tarsal organ on female tarsus I, dorsal view **14** trichobotrial base, female tarsus I, dorsal view **15–16** female palp and the claw at its tip (arrowed). Scale bars: 1 μm (**13–14**), 5 μm (**12**), 10 μm (**11, 15**), 50 μm (**10, 16**), 0.1 mm (**9**).

**Figures 17–24.**
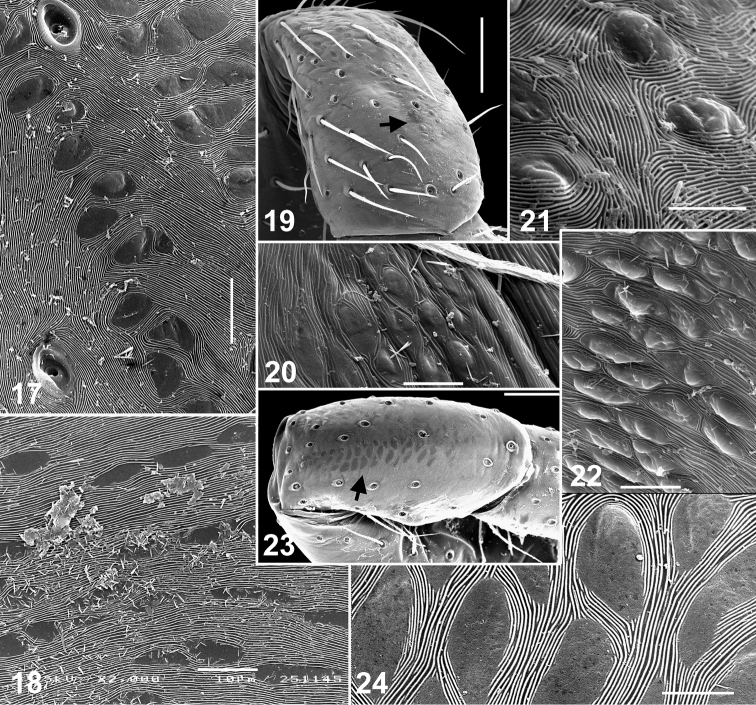
Skin structures of *Eupoa lehtineni* sp. n. (**17, 19–22**) and *Eupoa thailandica* sp. n. (**18, 23, 24**). **17** male patella I, dorsal view **18, 20, 22** female carapace, lateral view **19, 21, 23–24** female patella I, dorsal view. Scale bars: 5 μm (**21, 24**), 10 μm (**17–18, 20, 22**), 50 μm (**19, 23**).

Small to very small spiders ranging from about 1.65 to 2.45 mm in length. Sexes similar in general body form; sexual dimorphism is poorly marked and can be seen in the following characters: dorsal scutum presents in males (absent in females), body coloration in males is slightly darker or more contrastingly coloured, anterior and posterior pairs of spinnerets are of contrasting colours in males (brown/dark grey *vs.* yellow; e.g., *Eupoa prima*, *Eupoa yunnanensis*), and legs I and II in males are often with no spines or just with 2/3 pairs of ventral spines on metatarsi (always with spines on tibiae and metatarsi in females; e.g., *Eupoa schwendingeri* sp. n., *Eupoa yunnanensis*). *Carapace*: rather high, with abruptly declining, practically vertical thoracic part (Figs 6, 59, 90); sparsely covered with white elongated pinnate scales (Figs 1–3); fovea absent (Figs 73, 75); lateral sides of carapace, near ALEs, with vertical rows of skin structures (50-60 in a group) looking like either as rounded or elongated smooth bare patches, slightly risen above the surrounding skin (Figs 20, 22), or as flat elongated patches with what looks like several micro-pustules situated on them (Fig. 18); similar bare skin structures occur on leg patellae (see below; Figs 20–21). *Eyes*: in three rows, with large black areas around eyes (Figs 42–46, 58–59); anterior eye row wider in both sexes, so the quadrangle is as an inverted trapezium; second row midway between ALE and PLE; quadrangle length 52–66% of carapace length. *Clypeus*: narrow, about 17–47% of AME diameter (from frontal view; Figs 4–5), visibly backward sloping (Figs 6, 59). *Chelicerae*: small and vertical (Figs 4–5, 7; promargin with two small teeth; retromargin with three small teeth (Fig. 8). *Maxillae*: slightly convergent; usual shape. *Labium*: transverse-ovoid. *Sternum*: as inverted cone with swollen lateral sides (Figs 30, 72, 76). *Pedicel*: short, in live specimens not visible in dorsal view. *Abdomen*: elongate, covered with elongated pinnate scales (Fig. 32); dorsal scutum present in males; colour markings on dorsum simple, either consisting of a median yellow stripe (Figs 41, 43, 45) or two rows of spots with a pair of largest sports at the rear of dorsum (Figs 85–86). *Book-lung covers*: usual shape, not sclerotized. *Spinnerets*: posterior pair almost two times longer than anterior pair (Figs 31–32). *Legs*: subequally developed (Fig. 9); legs I in males usually with dark brown longitudinal stripes; trichobothrial bases relatively flat and striated (Fig. 14); tarsal organ as a rounded or ovoid pit (Figs 12–13); tarsal claws narrow, with poorly developed teeth (Figs 10–11); skin structures of two kinds present anteriorly on the dorsal surface of leg patellae, situated in a longitudinal row of about 20–30 pores (arrowed in Figs 19, 21, 23–24): structures of the first kind represents flat elongated and smooth bare patches with what looks like several micro-pustules situated on them (Figs 17, 24); structures of the second kind look like a rounded or elongated or circular smooth bare patch, slightly risen above the surrounding skin (Figs 21; similar bare structures occur on the carapace, see above, Figs 20, 22), these structures resemble the second kind of skin pores described in *Neon* (Logunov 1998: figs 7, 11). *Leg formula*: IV,I,III,II in both sexes, rare IV,I,II,III in females. *Leg spination*: in males legs I and II are often spineless (or with a few spines on Mt I: v 2–2–2ap); in females Tb I v 2–2–2ap, Tb II pr and rt 0–1–0, v 1–1/0 and Mt I and II v 2–2–2ap; in both sexes Tb III and IV usually pr and rt 0–1–0 (or 0–1). *Female palp*: general form; with an apical claw (arrowed in Figs 15–16). *Male palp*: swollen and relatively large for the size of the spiders (Figs 42–43); femur of usual shape, except for *Eupoa prima* (Figs 80–83), shorter than cymbium; patella swollen, with one (Figs 61, 70, 84, *etc.*) or two (Fig. 80) apophyses that sometimes are as long as the femur (*Eupoa prima*; Fig. 80) or poorly-developed and inconspicuous (*Eupoa lehtineni* sp. n.; Fig. 50), or sometimes bifid (Figs 96, 126); tibia shorter than patella, with one or two tibial apophyses (Figs 49, 59, 116, etc.) that sometimes are poorly-developed (*Eupoa prima*; Figs 81, 84) or covered with long hairs (*Eupoa thailandica* sp. n.; Fig. 111); cymbium well-developed, sometimes with bunches of white hairs at its basis (e.g., in *Eupoa thailandica* sp. n.; Figs 106–107); tegulum well-developed (sometimes on one side of the bulb only; e.g., in *Eupoa pappi* sp. n.; Figs 70–71) and usually with tegular apophysis (Figs 52, 60, etc.), which sometimes poorly-developed (e.g., in *Eupoa schwendingeri* sp. n.; Fig. 98) or even absent (e.g., in *Eupoa pulchella* sp. n.; Figs 91–96); median apophysis present (Figs 49, 83, etc.), but sometimes poorly-developed (e.g., in *Eupoa pappi* sp. n.; Figs 67–68); compound terminal apophysis present and situated inside the apical cavity of tegulum, either thin and long (Figs 52, 61) or strong, with a longitudinal groove on its anterior edge (e.g., in *Eupoa pappi* sp. n. or *Eupoa thailandica* sp. n.; Figs 66–68, 109); embolus usually very long and coiled, making 1.5–2 revolutions, with its terminal end resting on top of the cymbium (Figs 36, 51, 62), or can be short (Fig. 94) and even fingerlike and apically bifurcated in some species (*Eupoa yunnanensis*; Fig. 125). *Female copulatory organs*: simple, with a pair of copulatory openings that usually spaced up from each other and poorly visible on the epigynal plate; epigynal plate flat (Figs 29, 48, 100) or sometimes with a central shallow atrium (*Eupoa yunnanensis*; Fig. 127), usually covered with long light hairs (Figs 39–40); insemination ducts relatively short, directed to each other (Fig. 101; see also Żabka (1985: fig. 169) or being subparallel (Figs 53, 118); receptacles rounded or bean-shaped, usually much stronger sclerotized than insemination ducts (Figs 53, 118, 128); in most species receptacles and fertilization ducts are situated at the posterior end of the vulva (near the epigastric furrow), but sometimes lie at its anterior end (*Eupoa lehtineni* sp. n.; Figs 53–54).

**Figures 25–32.**
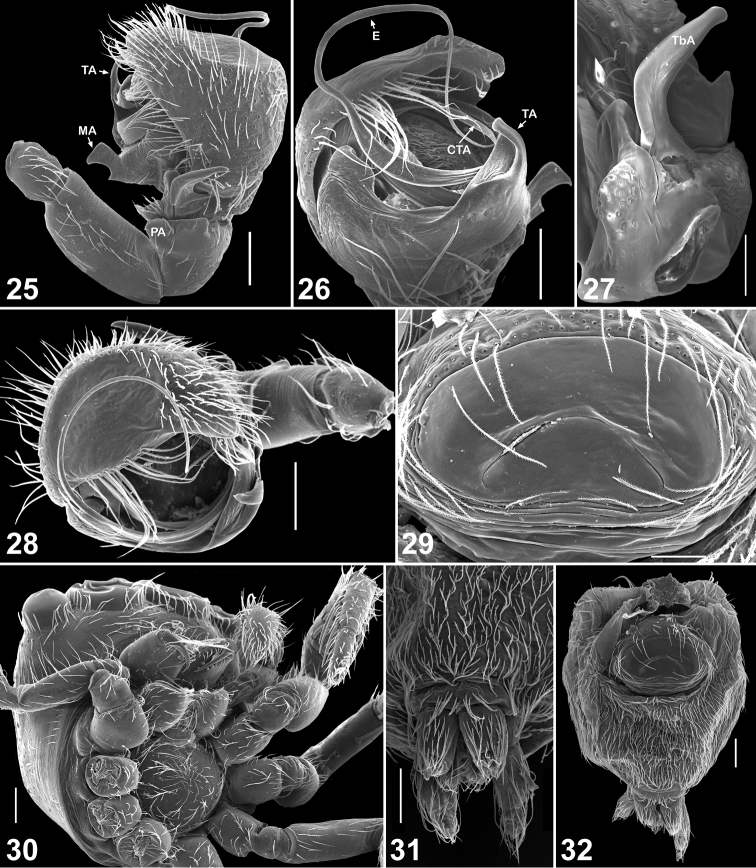
Copulatory organs and somatic characters of *Eupoa lehtineni* sp. n. **25** male palp, retrolateral view **26** ditto, median view **27** male tibial apophysis, retrolateral view **28** male palp, apical view **29** epigyne, ventral view **30** female carapace, ventral view **31** female spinnerets, ventral view **32** female abdomen, ventral view. Abbreviations as explained in ‘Material and methods’. Scale bars: 50 μm (**27, 29**), 0.1 mm (**25–26**, **28**, **30–32**).

#### Diagnosis and affinities.

Of the described salticid genera, *Eupoa* is closest to *Corusca* Zhou & Li, 2013 (10 species) and *Sinoinsula* Zhou & Li, 2013 (12 species) known from Hainan Island of China (Zhou and Li 2013a, b). *Eupoa* can vaguely be distinguished from *Corusca* by the presence of patellar apophysis in the males (except for *Corusca viriosa* Zhou & Li, 2013 and *Corusca wuzhishanensis* Zhou & Li, 2013) and the paired copulatory openings in the females. This is why none of the new species described in this paper has been assigned to *Corusca*, even *Eupoa lehtineni* sp. n. and *Eupoa lobli* sp. n. of which the conformation of male copulatory organs is very similar to that of the *Corusca* species. *Eupoa* can be distinguished from *Sinoinsula* by the conformation of copulatory organs in the males: viz., the presence of median apophysis and, more importantly, by the apical/medio-lateral origin of the embolus (prolateral in *Sinoinsula*). Nevertheless, despite the aforementioned differences it is likely that the three genera are not only closely related but could even be treated as one, under the name of *Eupoa*. The original differential diagnoses of *Corusca* and *Sinoinsula* (see Zhou and Li 2013a) were purely based on a few characters of the copulatory organs, some of which vary (e.g., the presence/absence of the patellar, tegular or compound terminal apophyses), whereas somatic morphology was largely neglected. It seems that at least the limits of *Corusca* are unclear and can be reconsidered in the future. In the opinion of one of us (DL), the genera *Corusca* and *Sinoinsula* would be better considered the subgenera of *Eupoa* (*s. lato*), unless additional reliable diagnostic characters in somatic morphology were found. However, no synonymy is being made at this time. This problem is outside the scope of the present paper and will be considered in more detail in the future.

The phylogenetic relationships of *Eupoa* remain poorly resolved. On the basis of two morphological characters (presence of the median apophysis and a tarsal claw in the female palp; Figs 15–16) and molecular data, Maddison et al. (2007) argued that the genus *Eupoa* is a basal salticid of which phylogenetic placement lies outside the Salticoidea, and even outside the Spartaeinae (as it lacks a recognised tegular furrow; *sensu* Wijesinghe 1992). Obviously, further studies are required to resolve the correct phylogenetic placement of *Eupoa*.

#### Composition.

Currently, 12 species are included in *Eupoa* (Platnick 2014; present data).

#### Distribution.

*Eupoa* seems to be restricted to the Oriental Region, for all the described species thereof as well as those of two closely related genera, *Corusca* and *Sinoinsula*, are restricted to SE Asia (Żabka 1985; Peng and Kim 1997; Maddison et al. 2007; Zhou and Li 2013a; present data).

### Descriptions

#### Eupoa daklak sp. n.

http://zoobank.org/EF8169C1-C2D9-45C3-BAC0-38DD65DBBD40

http://species-id.net/wiki/Eupoa_daklak

Figs 33–35

##### Type.

Holotype ♀ (ZMUM) from Viet-Nam, Dak Lak Prov., c. 25 km SSW of Buon Ma Thuot (c. 12°26'48"N, 107°58'E), Dak Linh, 500 m a.s.l., 28-29.04.1986, L.N. Medvedev & S.I. Golovatch.

##### Etymology.

The species epithet is a noun taken in apposition of the type locality: Daklak province of Viet-Nam.

##### Diagnosis.

**Figures 33–35.**
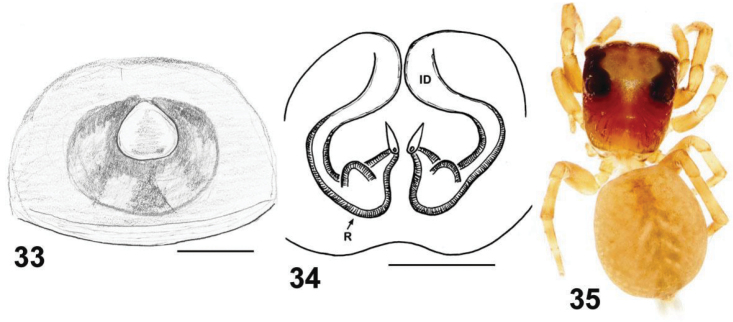
Copulatory organs and somatic characters of *Eupoa daklak* sp. n. **33** epigyne, ventral view **34** vulva, dorsal view **31** female body, dorsal view. Abbreviations as explained in ‘Material and methods’. Scale bars: 0.1 mm.

The central shallow depression of the epigyne of *Eupoa daklak* sp. n. (Fig. 33) is unique among all the *Eupoa* species known to us. The male of *Eupoa daklak* sp. n. remains unknown.

##### Comments.

Although this species is described on the basis of a single female, it is highly unlikely that it could belong to one of the three other new species described below on the basis of males (*Eupoa lobli* sp. n. from Malaysia; *Eupoa pappi* sp. n. and *Eupoa pulchella* sp. n. from Thailand). All *Eupoa* species, as well as those of two closely related genera (*Corusca* and *Sinoinsula*), are litter-dwellers, with most of them (except for *Eupoa lehtineni* sp. n.) having small, local ranges. For instance, 22 distinct species of *Corusca* and *Sinoinsula* were recently described from Hainan Island of China alone (Zhou and Li 2013a, b). Therefore, it is safe to conclude that this and three other *Eupoa* species that are described herein on the basis of a single sex from very distant localities indeed belong to different species. Finally, there is no technical way to match this female with one of the males, unless they were collected together.

##### Distribution.

The type locality only.

##### Description.

Male unknown.

FEMALE. *Measurements*. Carapace 0.89 long, 0.76 wide and 0.49 high at PLE. Ocular area 0.59 long, 0.77 wide anteriorly and 0.71 wide posteriorly. Diameter of AME 0.24. Clypeus height 0.07, chelicera length 0.29. Abdomen 1.06 long, 0.89 wide. Length of leg segments: I: 0.47 + 0.22 + 0.29 + 0.29 + 0.19; II: 0.41 + 0.19 + 0.23 + 0.23 + 0.19; III: 0.39 + 0.17 + 0.26 + 0.27 + 0.19; IV: 0.61 + 0.23 + 0.43 + 0.34 + 0.23. *Leg spination*. Leg I: Tb v 2-2-2ap; Mt v 2-2-2ap. Leg II: Tb v 2-2; Mt v 2-2-2ap. Leg III: Tb pr and rt 0-1-0; Mt pr 1-1ap, rt and v 1-0. Leg IV: Tb pr and rt 0-1-0; Mt pr 1-0-2ap, rt 1-0-1ap, v 1-0. *Coloration* (Fig. 35). Carapace yellow-brown, with brownish radial veins on thorax and yellow eye field. Blackened around eyes. Clypeus naked, yellow, with a brown marginal line. Sternum, maxillae, labium and chelicerae yellow, tinged with brown. All abdomen brownish yellowish, without a colour pattern. Book-lung covers yellow, tinged with brown. Spinnerets: anterior pair brownish, posterior pair pale yellow. All legs yellow, with poorly marked brownish patches at segment joints. Palps: femora and patellae brownish, metatarsi and tarsi yellow. Epigyne and vulva as in Figs 33–34: epigynal plate as a rounded shallow depression; insemination ducts thin and transparent; pear-shaped receptacles are close to each other, meeting up along the median line.

#### Eupoa lehtineni sp. n.

http://zoobank.org/ACCC403B-039D-4B0E-8EE1-4C9AA4458999

http://species-id.net/wiki/Eupoa_lehtineni

Figs 1 –11 , 13 –17 , 19–22 , 25–32 , 36 [Fig F7] –54

##### Types.

Holotype ♂ (ZMTU) from India, Meghalaya, East Khasi Hills, Umran, riverside forest, 1200 m a.s.l., 4.05.1979, P.T. Lehtinen.

Paratypes: INDIA: 2♂5♀ (ZMTU), together with the holotype. – THAILAND: 3♂5♀ (MHNG, one male without abdomen and palp), Trat Prov., Ko Chang, west side (12°03'N, 102°18'E), WINKLER-extraction in secondary forest with primary spots (AS-T-5), 50-200 m a.s.l., 3-23.12.1999, A. Schultz; 1♀ (MHNG), Kanchanaburi Prov., Sai Yok Distr., Sai Yok National Park, near HQ, 100 m a.s.l., 21.07.1987, P.J. Schwendinger. – VIET-NAM: 1♀ (ZMUM), Dong Nai Prov., Vinh Cuu Dist., Vinh Cuu Nature Reserve (= Ma Da Forest), c. 6 km N of Ba Hao Vil. (c. 11°19'N, 107°05'E), 80 m a.s.l. (sample-5), 10.05.1995, T. Sergeeva; 1♀ (ZMUM), same locality (sample-11), 6.06.1995, T. Sergeeva.

##### Etymology.

The species is named in honour of our colleague and friend, Dr Pekka Lehtinen (Turku, Finland), who collected the holotype.

##### Diagnosis.

By the conformation of the large, round tegulum, the tibial apophysis and the thin compound terminal apophysis that is hidden in the apical cavity of tegulum (Figs 26, 52), the male of *Eupoa lehtineni* sp. n. is most similar to that of *Eupoa lobli* sp. n. (Figs 60–62), but can easily be distinguished by the shape of tegular and median apophyses. The female of *Eupoa lehtineni* sp. n. has the easily recognizable triangle epigynal plate (Figs 29, 39–40) and the mutual arrangement and shape of receptacles (Figs 53–54).

##### Distribution.

From eastern India, south-eastward to Thailand and southern Viet-Nam (present data).

##### Description.

**Figures 36–40.**
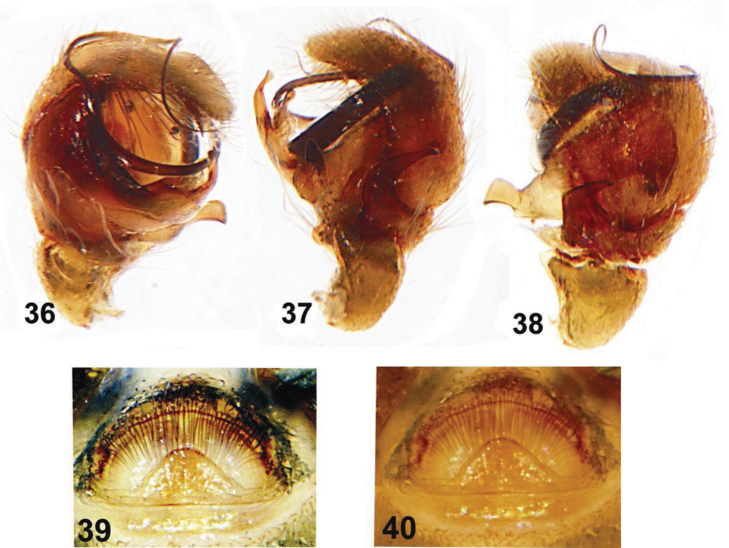
Copulatory organs of *Eupoa lehtineni* sp. n. from India. **36** male palp, median view **37–38** ditto, retrolateral view **39–40** epigyne, ventral view.

MALE (from Thailand: Ko Chang). *Measurements*. Carapace 0.94 long, 0.79 wide and 0.51 high at PLE. Ocular area 0.61 long, 0.79 wide anteriorly and 0.77 wide posteriorly. Diameter of AME 0.24. Clypeus height 0.06, chelicera length 0.29. Abdomen 0.76 long, 0.58 wide. Length of leg segments: I: 0.44 + 0.22 + 0.30 + 0.26 + 0.16; II: 0.40 + 0.21 + 0.21 + 0.24 + 0.19; III: 0.38 + 0.18 + 0.22 + 0.27 + 0.20; IV: 0.59 + 0.23 + 0.41 + 0.30 + 0.23. *Leg spination*. Leg I: Mt v 2-2-2ap. Leg II with no spines. Leg III: Tb pr and rt 0-1-0. Leg IV: Tb pr and rt 0-1-0; Mt pr and rt 0-1-1-ap. *Coloration* (Figs 42–43). Carapace light grey-brown, with grey-yellow eye field and with poorly visible dark brown radial veins on thorax. Blackened around eyes. Clypeus naked, yellow-brown. Sternum light grey-brown. Maxillae, labium and chelicerae grey-yellow. Abdomen light grey-brown, dorsum completely covered with large scutum and with a thin longitudinal, medial yellow line. Book-lung covers light grey-brown. Spinnerets: anterior pair grey brownish, posterior pair yellow. All legs grey-yellow, with dark brown patches at segment joints, but patellae, tibiae and metatarsi I pro-ventrally with dark brown longitudinal stripe. Palp grey-yellow, its structure as in Figs 25–28, 36–38, 49–52: patellar apophysis short and obtuse; tibial apophysis blade-shaped, bent dorsad; tegulum well-developed, apically with prominent, hook-shaped tegular apophysis; median apophysis massive, directed ventrad; compound terminal apophysis thin and long, situated inside the apical cavity of tegulum; embolus coiled, making two revolutions, with its terminal end resting on top of the cymbium.

**Figures 41–46. F7:**
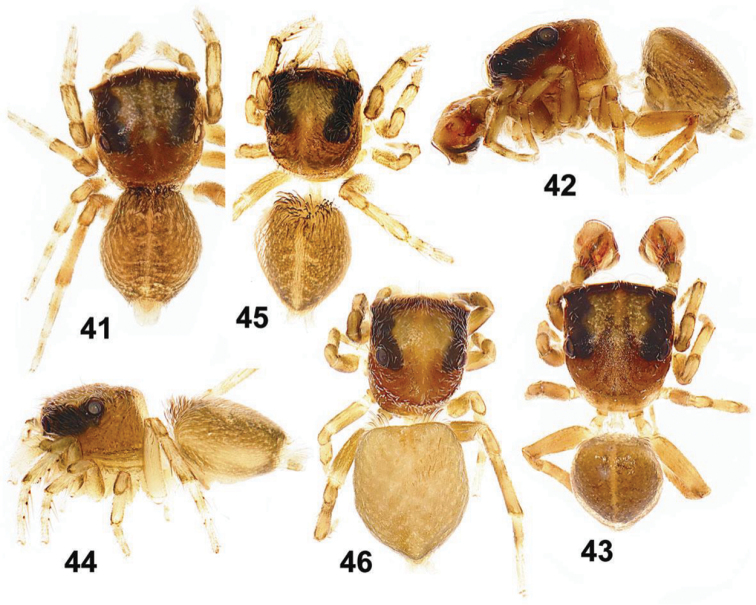
General appearance of *Eupoa lehtineni* sp. n. **41**, **45–46** females, dorsal view **42** male, lateral view **43** ditto, dorsal view **44** female, lateral view. Specimens: **41–43** – India; **44–46** – Viet-Nam.

**Figures 47–54.**
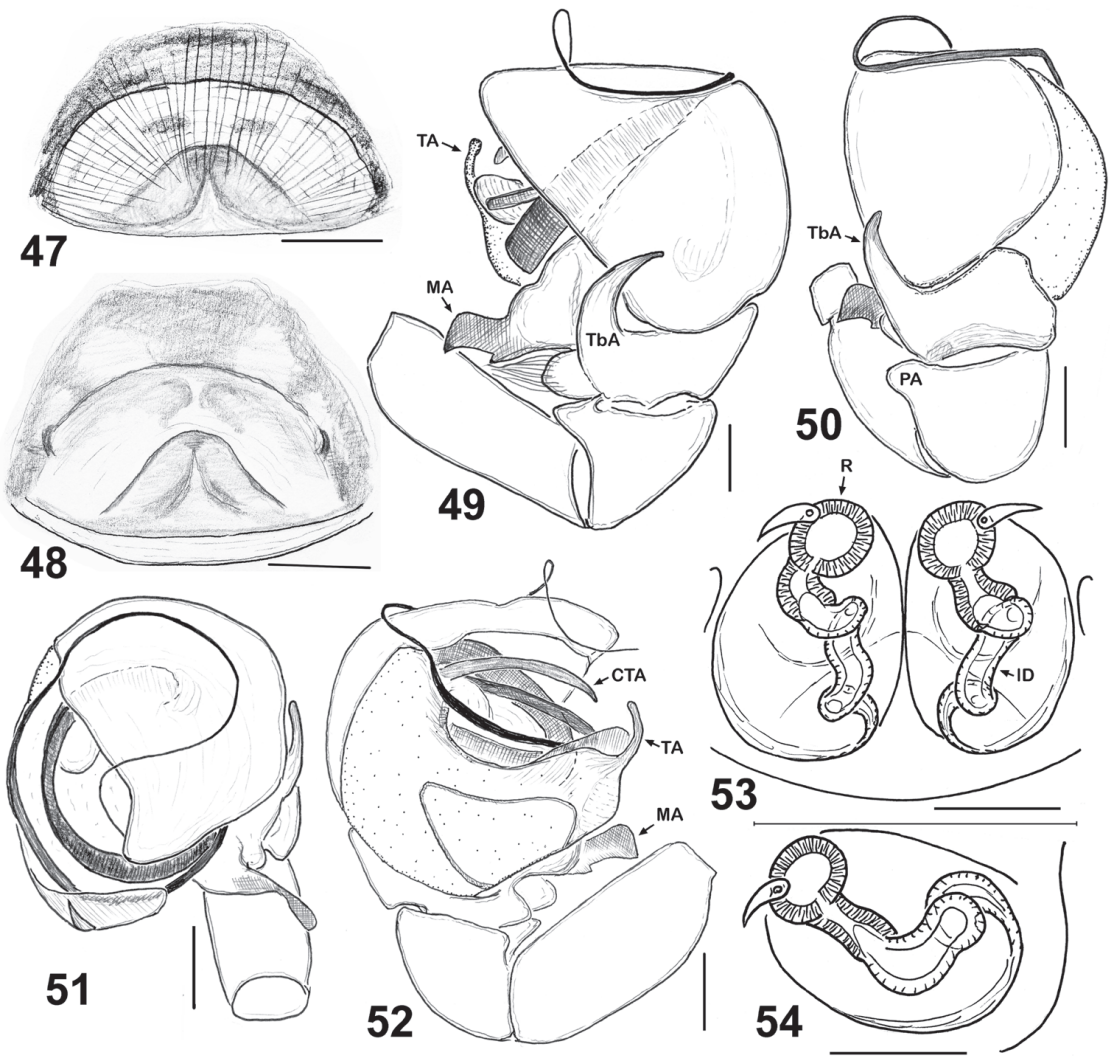
Copulatory organs of *Eupoa lehtineni* sp. n. **47–48** epigyne, ventral view **49** male palp, retrolateral view **50** ditto, dorsal view **51** ditto, apical view **52** ditto, median view **53–54** vulva, dorsal view. Abbreviations as explained in ‘Material and methods’. Specimens: **49–53** – India; **47–48**, **54**– Viet-Nam. Scale bars: 50 μm (**27, 29**), 0.1 mm (**25–26**, **28**, **30–32**).

FEMALE (from Viet-Nam: Dong-Nai). *Measurements*. Carapace 0.93 long, 0.73 wide and 0.51 high at PLE. Ocular area 0.59 long, 0.75 wide anteriorly and 0.71 wide posteriorly. Diameter of AME 0.24. Clypeus height 0.06, chelicera length 0.33. Abdomen 0.89 long, 0.71 wide. Length of leg segments: I: 0.49 + 0.26 + 0.31 + 0.31 + 0.21; II: 0.39 + 0.21 + 0.23 + 0.26 + 0.18; III: 0.39 + 0.17 + 0.24 + 0.31 + 0.21; IV: 0.63 + 0.23 + 0.49 + 0.37 + 0.23. *Leg spination*. Leg I: Tb v 2-2-2ap; Mt v 2-2-2ap. Leg II: Tb pr 0-1-0, v 1-1; Mt v 2-2-2ap. Leg III: Tb pr 0-1-0, Mt pr 1-1ap, rt 1ap. Leg IV: Tb pr and v 0-1-0; Mt pr 2-0-2ap, rt 1-0-1ap. *Coloration* (Figs 41, 44–46). Carapace pale brown, with yellow eye field and a thin longitudinal median line on thorax. Blackened around eyes. The entire carapace covered (not very densely) with white and transparent elongated scales. Clypeus naked, brown-yellow. Sternum yellow, tinged with brown. Maxillae, labium and chelicerae pale yellow. Abdomen: dorsum light brown, with a longitudinal medial yellow line; sides light brown, each with a wide yellow stripe; venter yellow. Anterior edge of carapace with a bunch of long brownish hairs. Book-lung covers yellow. Spinnerets: anterior pair brownish, posterior pair pale yellow. All legs yellow, with brownish (semi)rings at segment joints, but femora, patellae and tibiae I pro-ventrally with dark brown stripe. Palps: femora and patellae light brown, metatarsi and tarsi yellow. Epigyne and vulva as in Figs 29, 39–40, 47–48, 53–54: epigynal plate triangular, with obtuse tip directed anteriorly, covered with long whitish hairs; insemination ducts relatively short, subparallel and directed anteriorly; receptacles relatively small and rounded; receptacles and fertilization ducts situated in the anterior part of vulva.

#### Eupoa lobli sp. n.

http://zoobank.org/C5D9756D-9195-4014-A068-939D89C19F09

http://species-id.net/wiki/Eupoa_lobli

Figs 55 –65

##### Type.

MALAYSIA: 1♂ (MHNG, both palps detached), West Malaysia, Pahang, Cameron Highlands, 1520 m a.s.l., trail 14, Bukit Mentiga (tamisage), 23.03.1993, I. Löbl & F. Calame.

##### Etymology.

The species is named after the famous coleopterist, Dr. Ivan Löbl (Geneva, Switzerland), who collected the holotype.

##### Diagnosis.

The male of *Eupoa lobli* sp. n. is most similar to that of *Eupoa lehtineni* sp. n., but can easily be distinguished by the shape of tegular and median apophyses (cf. Figs 60–61 and 52). The female of *Eupoa lobli* sp. n. remains unknown.

##### Distribution.

The type locality only.

##### Description.

**Figures 55–59.**
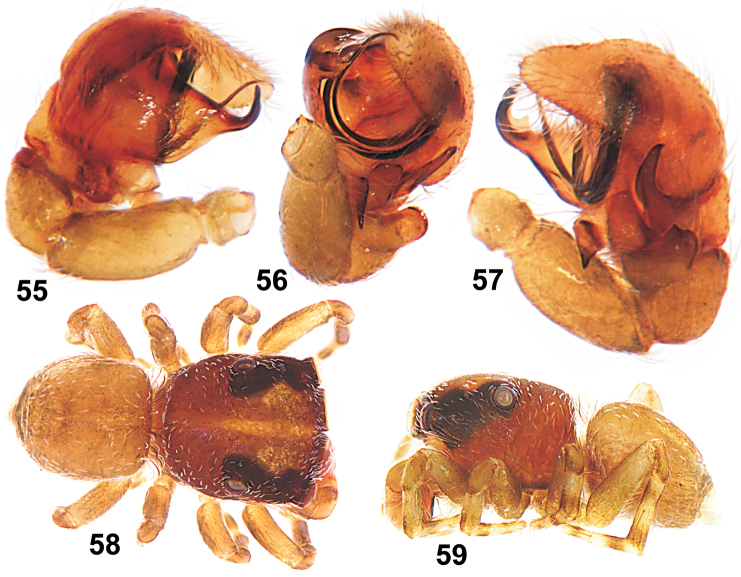
Copulatory organs and somatic characters of *Eupoa lobli* sp. n. (the holotype). **55** male palp, median view **56** ditto, apical view **57** ditto, retrolateral view **58** male general appearance, dorsal view **59** ditto, lateral view.

**Figures 60–65.**
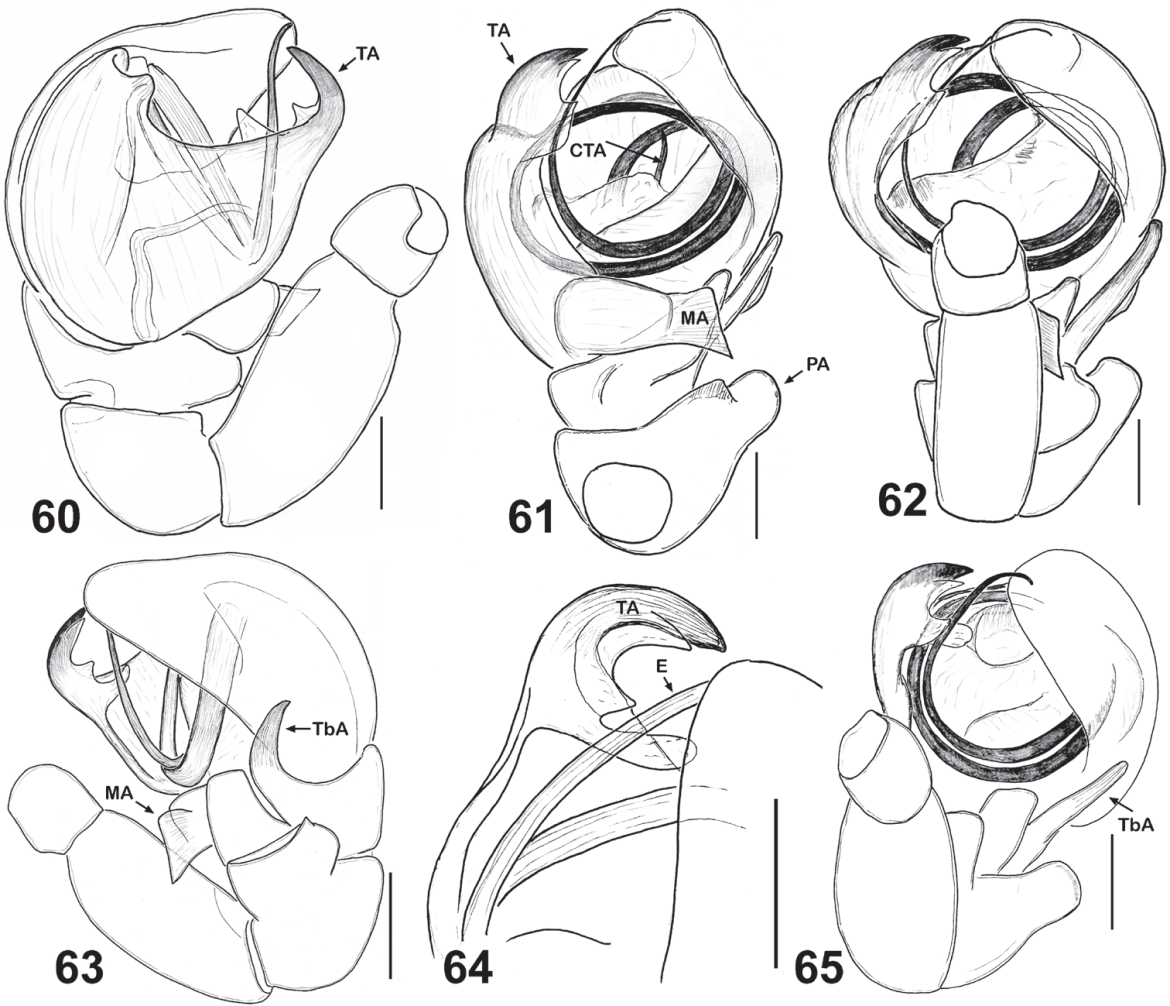
Copulatory organs of *Eupoa lobli* sp. n. (the holotype). **60** male palp, median view **61–62, 65** ditto, ventral view **63** ditto, retrolateral view **64** tegular apophysis, retrolateral view. Abbreviations as explained in ‘Material and methods’. Scale bars: 0.1 mm.

MALE. *Measurements*. Carapace 0.93 long, 0.74 wide and 0.51 high at PLE. Ocular area 0.54 long, 0.74 wide anteriorly and 0.67 wide posteriorly. Diameter of AME 0.23. Clypeus height 0.11, chelicera length 0.23. Abdomen 0.71 long, 0.64 wide. Length of leg segments: I: 0.46 + 0.21 + 0.31 + 0.29 + 0.21; II: 0.38 + 0.23 + 0.22 + 0.26 + 0.20; III: 0.37 + 0.19 + 0.21 + 0.22 + 0.20; IV: 0.64 + 0.22 + 0.39 + 0.31 + 0.25. *Leg spination*. Leg I: Tb v 0-1-0; Mt v 2-2-2ap. Leg II with no spines. Leg III: Tb rt 1-0. Leg IV: Tb pr and rt 0-1-0; Mt pr 1-0-1ap, rt 1-0-2ap. *Coloration* (Figs 58–59). Carapace light brown, sparsely covered with white elongated scales; eye field with a median yellow stripe. Blackened around eyes. Clypeus naked, yellow. Sternum, maxillae and labium yellow brownish. Abdomen light brown, with no colour pattern; dorsum completely covered with scutum. Book-lung covers light brown. Spinnerets: anterior pair brownish, posterior pair pale yellow. All legs yellow, with brownish patches at segment joints, but tibiae I and II pro-ventrally with dark brown longitudinal stripe. Palps brownish yellow, their structure as in Figs 55–57, 60–65: patellar apophysis short and wide, as if cut on its tip; tibial apophysis claw like, blade-shaped and directed dorsad; tegulum well-developed, with prominent, wide tegular apophysis apically possessing three hook-shaped, obtuse teeth; median apophysis massive, directed latero-ventrad; compound terminal apophysis relatively short and thin, hidden inside the cavity formed by cymbium and tegulum; embolus coiled, making two revolutions, with its tip resting on top of the cymbium.

Female unknown.

#### Eupoa pappi sp. n.

http://zoobank.org/72FED4F4-8221-4D13-9ABE-726088FDF2A5

http://species-id.net/wiki/Eupoa_pappi

Figs 66–71

##### Type.

Holotype ♂ (HNHM) from Thailand Trang Prov., Thung Khai Botanic Garden, primary lowland rainforest, 12.11.2004, L. Papp & M. Földvári.

##### Etymology.

The species is named after Dr. László Papp (Budapest, Hungary), who collected the holotype.

##### Diagnosis.

By having the massive compound terminal apophysis with a deep longitudinal groove on its apical edge (Fig. 67), the male of *Eupoa pappi* sp. n. is most similar to that of *Eupoa thailandica* sp. n. (Figs 109, 115), from which it differs in having the larger open cavity of tegulum and the shape of tegular and compound terminal apophyses. The female of *Eupoa pappi* sp. n. remains unknown.

**Figures 66–71.**
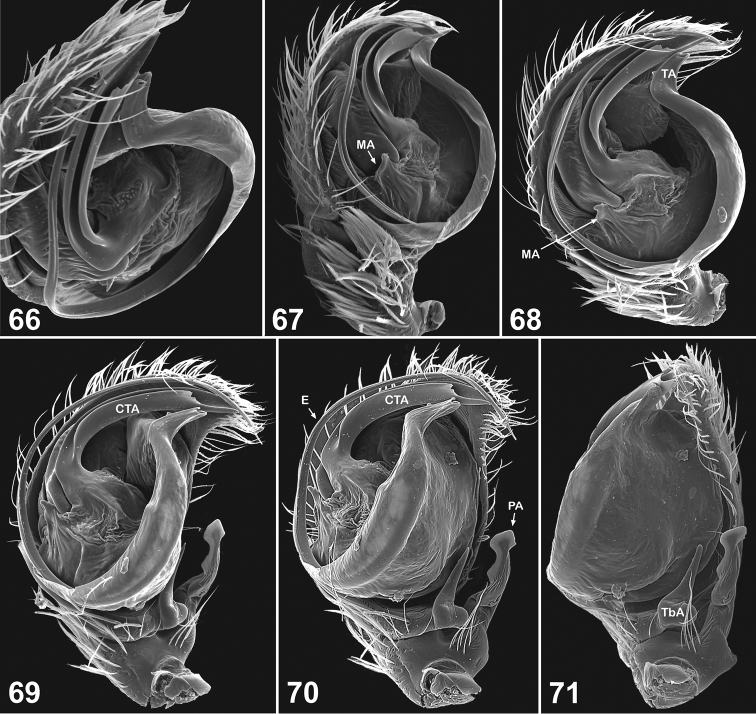
Copulatory organs of *Eupoa pappi* sp. n. (the holotype). **66** male palp, apical view **67–68** ditto, median view **69** ditto, ventral view **70–71** ditto, retrolateral view. Abbreviations as explained in ‘Material and methods’.

##### Distribution.

The type locality only.

##### Description.

MALE (one palp missing). *Measurements*. Carapace 0.93 long, 0.75 wide and 0.60 high at PLE. Ocular area 0.60 long, 0.80 wide anteriorly and 0.63 wide posteriorly. Diameter of AME 0.23. Clypeus height 0.10, chelicera length 0.28. Abdomen 0.88 long, 0.63 wide. Length of leg segments: I: 0.55 + 0.24 + 0.40 + 0.39 + 0.20; II: 0.49 + 0.21 + 0.29 + 0.29 + 0.17; III: 0.48 + 0.33 + 0.34 + 0.35 + 0.23; IV: 0.78 + 0.28 + 0.53 + 0.43 + 0.28. *Leg spination*. Leg I: Mt v 2-2ap. Leg II and III: no spines. Leg IV: Tb pr and rt 0-1-0; Mt pr and rt 1-1ap. *Coloration*. Carapace light yellow, with brownish margins. Eye field brownish, blackened around eyes. Clypeus naked, yellowish brownish. Sternum, maxillae, labium and chelicerae light yellow. Abdomen: dorsum yellow, with dark grey longitudinal lateral bands and two round, large dark grey spots in its rear part; venter yellow. Book-lung covers yellow. Spinnerets: anterior pair yellow, posterior pair dark grey. All legs yellow, but patellae IV with brownish sides. Palps: femora yellow, remaining segments brownish yellow. Palpal structure as in Figs 66–71: patellar apophysis long, reaching almost a half of the cymbial lenght; tibia with a median bunch of thick white hairs; tibial apophysis sharpened at its tip directed anteriorly and with a wide, rounded base; tegulum flat and developed on its pro-lateral side only; tegular apophysis situated on the apical end of tegulum and bent laterad; median apophysis poorly developed, seen as a short triangle process at the base of compound terminal apophysis (but separated from it by membrane); compound terminal apophysis massive, with a deep longitudinal groove on its apical edge; embolus whip-shaped, making one revolution.

Female unknown.

#### Eupoa prima

Żabka, 1985

http://species-id.net/wiki/Eupoa_prima

Figs 72 –84

Eupoa prima Żabka, 1985: 220, figs 161–169 (D♂♀).

##### Types.

Holotype ♂ (ZMTU) from Viet-Nam, Bac Thai, Bach Tong, Duong Quang (apparently, Bach Thong Distr., Bac Kan Prov.), jungle slope, 900 m a.s.l., 17.10.1978, P.T. Lehtinen.

Paratypes: VIET-NAM: 1♂1♀ (ZMTU; epigyne missing), together with the holotype; 1♀ 5juv. (HNHM; epigyne missing), c. 5 km E of Lao Cai town, 200 m a.s.l., sieving forest litter, 1971, Topál-Matskásl.

##### Diagnosis.

Both sexes of *Eupoa prima* are most similar to those of *Eupoa jingwei* and *Eupoa nezha* known from Guangxi province of China; threes species seem to form a natural species group. Males of all three species can easily be separated by the shape of tegular and patellar apophyses; cf. Figs 80–84 with figs 1–3, 8–10 in Maddison et al. (2007). Females of these three species cannot be readily distinguished now as they have the virtually identical conformation of their epigynes; cf. fig. 167 in Żabka (1985) and Figs 7, 13 in Maddison et al. (2007), whereas the vulvas of *Eupoa jingwei* and *Eupoa nezha* has not been studied and illustrated yet.

##### Comments.

Unfortunately, neither the ♀ allotype deposited at the ZMTU, nor the ♀ paratype deposited at the HNHM possesses its epigyne, although in both cases there are separate micro-vials that should have contained them. Thus, our notion about the epigynal and vulval structures of *Eupoa prima* is based on the original illustrations by Żabka (1985: figs 167–169).

##### Distribution.

Southern Viet-Nam (Żabka 1985).

##### Description.

**Figures 72–77.**
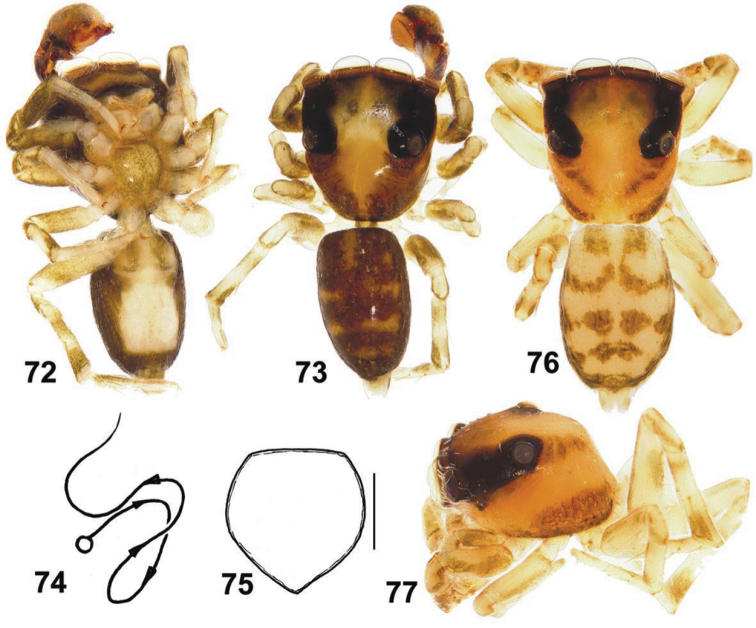
General appearance and somatic characters of *Eupoa prima* (♂ holotype and ♀ allotype). **60** male body, ventral view **73** ditto, dorsal view **74** diagrammatic course of the embolar path **75** female sternum, ventral view **76** female body, dorsal view **77** female carapace, lateral view. Scale bars: 0.25 mm (**76**).

**Figures 78–84.**
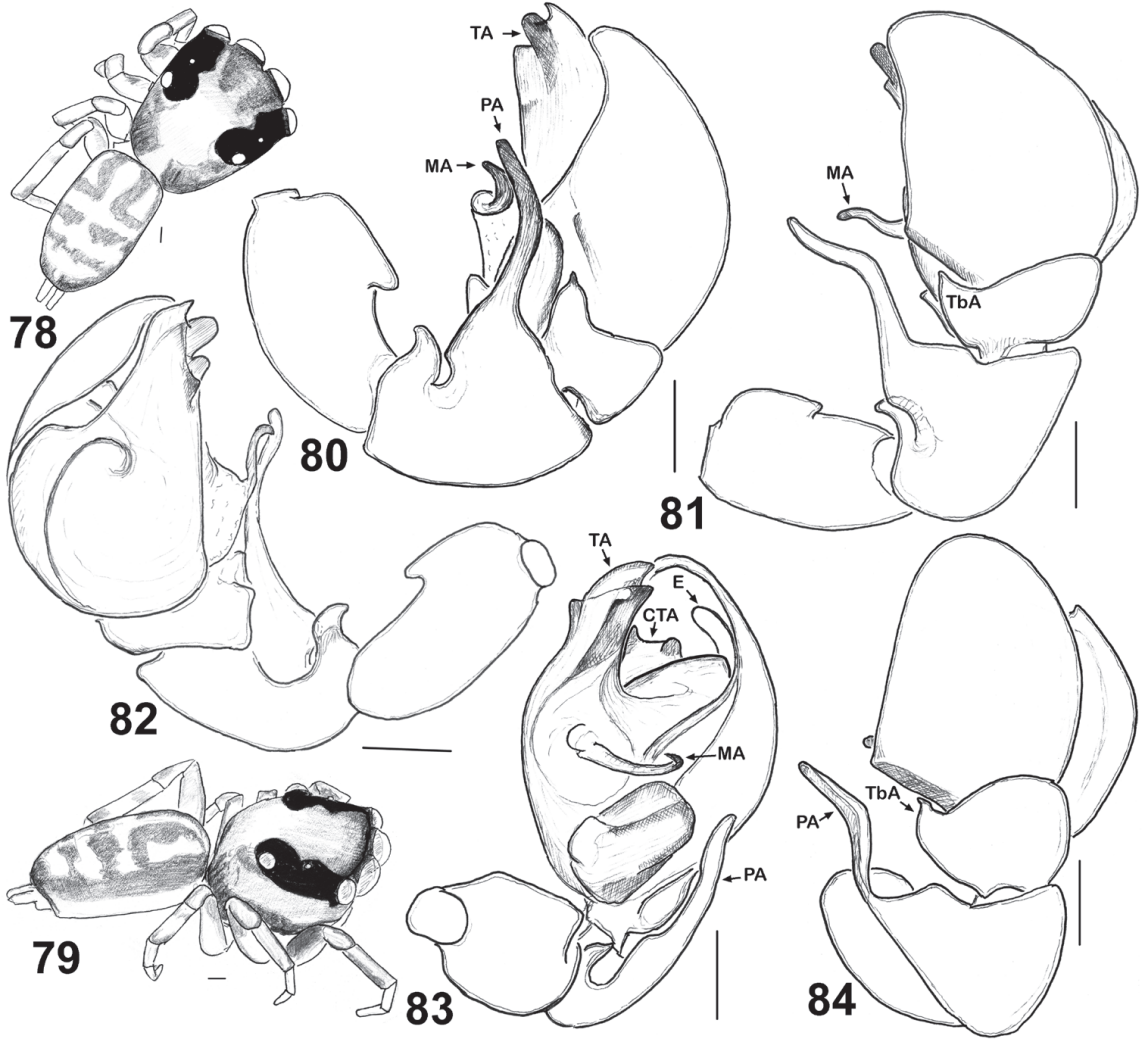
General appearance and copulatory organs of *Eupoa prima* (♂ paratype). **78** male body, dorsal view **79** ditto, lateral view **80** male palp, retrolateral view **81, 84** ditto, dorsal view **82** ditto, median view **83** ditto, ventral view. Abbreviations as explained in ‘Material and methods’. Scale bars: 0.1 mm.

MALE (the holotype). *Measurements*. Carapace 1.00 long, 0.89 wide and 0.55 high at PLE. Ocular area 0.64 long, 0.89 wide anteriorly and 0.81 wide posteriorly. Diameter of AME 0.29. Clypeus height 0.06, chelicera length 0.24. Abdomen 0.95 long, 0.61 wide. Length of leg segments: I: 0.54 + 0.26 + 0.34 + 0.36 + 0.19; II: 0.47 + 0.21 + 0.30 + 0.32 + 0.19; III: 0.46 + 0.21 + 0.27 + 0.35 + 0.23; IV: 0.71 + 0.30 + 0.51 + 0.39 + 0.29. *Leg spination*. Leg I: Tb v 1-1-1; Mt v 2-2-2ap. Leg II: Mt v 1-1-1ap. Leg III: Tb pr and rt 0-1. Leg IV: Tb pr 0-1; Mt pr and rt 1ap, v 0-1-0. *Coloration* (Figs 72–73, 78–79). Carapace yellow-brown, with a large yellow spot occupying almost the entire eye field; blackened around eyes. Clypeus naked, yellow, with a dark brown marginal line. Sternum, maxillae and labium yellow, tinged with brown. Chelicerae yellow, each with an anterior longitudinal brown stripe. Abdomen: dorsum brown, with shining scutum and a poorly marked yellow spot; sides brown; venter yellow. Book-lung covers yellow. Spinnerets: anterior pair brownish, posterior pair yellow. All legs yellow, but pro- and retrolateral sides of all segments (except for tarsi) brownish. Palps yellow-brown. Palpal structure as in Figs 74, 80–84: femur modified, with a wide proximal-ventral protuberance (as a short apophysis); patella with two apophyses, short basal apophysis and long median one, reaching almost a third of the cymbial lenght; tibial apophysis bi-ramous: its ventral branch wide, massive and visibly sclerotized and its dorsal branch short and cone-shaped; cymbium with triangular lobe in retrobasal part; tegulum well-developed; tegular apophysis situated on the median side of tegulum and directly anteriroly; median apophysis thin and hook-shaped, directed laterad; compound terminal apophysis low and wide, situated at the embolic base; embolus whip-shaped, making one revolution, its tip is resting on the dorsal side of tegular apophysis (its course is shown in Fig. 74).

FEMALE (the paratype). *Measurements*. Carapace 1.21 long, 1.01 wide and 0.60 high at PLE. Ocular area 0.76 long, 1.07 wide anteriorly and 0.96 wide posteriorly. Diameter of AME 0.36. Clypeus height 0.06, chelicera length 0.29. Abdomen 1.23 long, 0.86 wide. Length of leg segments: I: 0.70 + 0.30 + 0.43 + 0.43 + 0.21; II: 0.57 + 0.28 + 0.33 + 0.37 + 0.23; III: 0.56 + 0.21 + 0.36 + 0.43 + 0.27; IV: 0.89 + 0.29 + 0.64 + 0.56 + 0.31. *Leg spination*. Leg I: Tb v 2-2-2ap; Mt v 2-2-2ap. Leg II: Tb pr 0-1, v 1-1; Mt v 2-2-2ap. Leg III: Tb pr and rt 0-1-0; Mt pr 1-2ap, rt 2ap, v 1-0. Leg IV: Tb pr and rt 0-1-0, v 1-0; Mt pr 1-2ap, rt 1-1ap, v 1-0. *Coloration* much lighter than in the male (Figs 75, 77). Carapace yellow, with two wide brown stripes on sides. Blackened around eyes. Clypeus and ‘cheeks’ naked, yellow. Sternum, maxillae, labium and chelicerae yellow. Abdomen: dorsum yellow, with four transverse brown bands; sides yellow, each with a longitudinal brown stripe; venter yellow. Book-lung covers and spinnerets yellow. Legs I yellow, with pro- and retrolateral sides of femora, patellae and tibiae brownish. Legs II-IV yellow, with brownish patches at segment joints. Palps yellows, tinged with brown, but tarsi entirely yellow. Epigyne and vulva as in Żabka (1985: figs 167–169): epigynal plate flat, of the shape of inverted trapezium; paired copulatory openings spaced up; insemination ducts narrow and weakly sclerotized, directed to each other; receptacles sclerotized, bean-shaped.

#### Eupoa pulchella sp. n.

http://zoobank.org/DB59AEEB-D36B-4440-AF08-79C284E997C1

http://species-id.net/wiki/Eupoa_pulchella

Figs 87–88 , 91–96

##### Type.

Holotype ♂ (MHNG, both palps separated) from Thailand, Prov. Chiang Mai, Chiang Dao Distr., Doi Chiang Dao Wildlife Sanctuary, 510 m a.s.l., pitfall traps, 23.12.1990–15.01.1991, P. J. Schwendinger.

Paratypes: THAILAND: 3♂ (MHNG; palps of all specimens are separated), together with the holotype; 1♂ (MHNG; with the single detached palp), same locality, 510 m a.s.l., pitfall traps, 25.10–23.11.1990, P. J. Schwendinger.

##### Etymology.

From the Latin word ‘*pulchellus*’ meaning ‘pretty’.

##### Diagnosis.

This species differs from all *Eupoa* species known to us in having all the bulbal sclerites in apical position, as if grouped together at the top of tegulum (Figs 91–96), and the unique shape of patellar apophysis, which is short, thick and bi-ramous (Figs 93, 96). The female of *Eupoa pulchella* sp. n. remains unknown.

##### Distribution.

The type locality only.

##### Description.

MALE (♂ paratype with the palp). *Measurements*. Carapace 0.94 long, 0.73 wide and 0.46 high at PLE. Ocular area 0.56 long, 0.75 wide anteriorly and 0.70 wide posteriorly. Diameter of AME 0.23. Clypeus height 0.06, chelicera length 0.20. Abdomen 0.76 long, 0.59 wide. Length of leg segments: I: 0.44 + 0.21 + 0.29 + 0.29 +0. 19; II: 0.39 + 0.19 + 0.23 + 0.24 + 0.17; III: 0.37 + 0.16 + 0.23 + 0.26 + 0.27; IV: 0.59 + 0.23 + 0.41 + 0.33 + 0.21. *Leg spination*. Leg I: Tb v 2-2-2ap; Mt v 2-2ap. Leg II without spines. Leg III: Tb pr and rt 0-1-0. Leg IV: Tb pr and rt 0-1-1; Mt pr 1-2ap, rt 1-1ap. *Coloration*. Carapace brown, with a wide median yellow stripe started from the middle part of eye field and running to thorax; blackened around eyes. Clypeus naked and brown. Sternum, maxillae, labium and chelicerae pale yellow. Abdomen: dorsum brown, with two longitudinal rows of large yellow spots; sides brown; venter yellow. Book-lung covers yellowish brownish. Spinnerets: anterior pair brownish, posterior pair pale yellow. All legs yellow, with brown patches at segment joints, but femora, patellae and tibiae pro-ventrally and retrolaterally brown. Palps dark brown. Palpal structure as in Figs 91–96: patellar apophysis short, thick and bi-ramous; tibial apophysis short and cone-shaped; tegulum well-developed; tegular apophysis not developed; median apophysis fingerlike, directed anteriorly; compound terminal apophysis wide, plate-shaped and rugose; embolus short, S-shaped.

**Figures 85–90.**
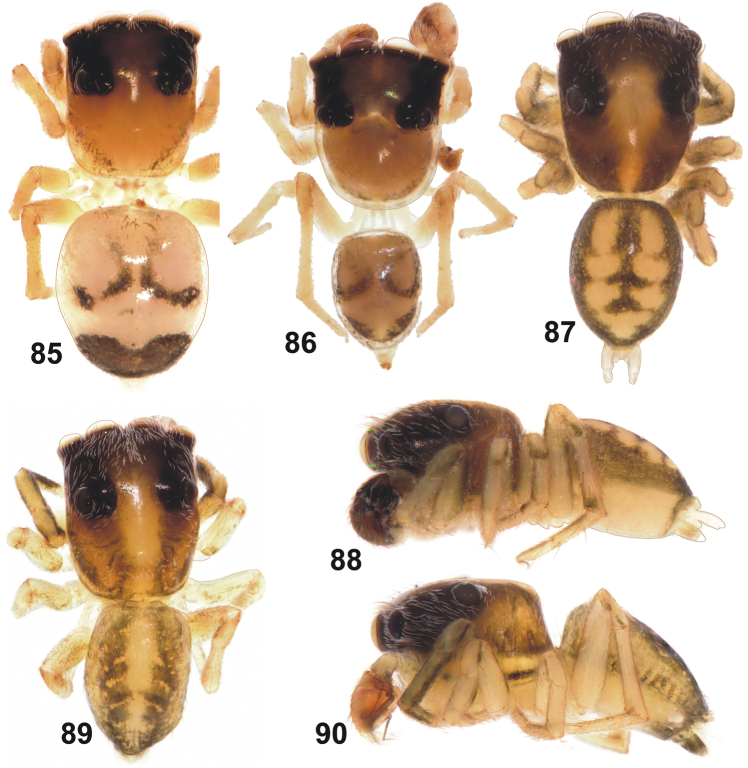
General appearance of *Eupoa pulchella* sp. n. (**87–88**, ♂ paratype), *Eupoa schwendingeri* sp. n. (♂ holotype, **89–90**) and *Eupoa thailandica* sp. n. (♀ and ♂ paratypes, **85–86**). **85** female body, dorsal view **86–87, 89** male body, dorsal view **88, 90** ditto, lateral view.

**Figures 91–96.**
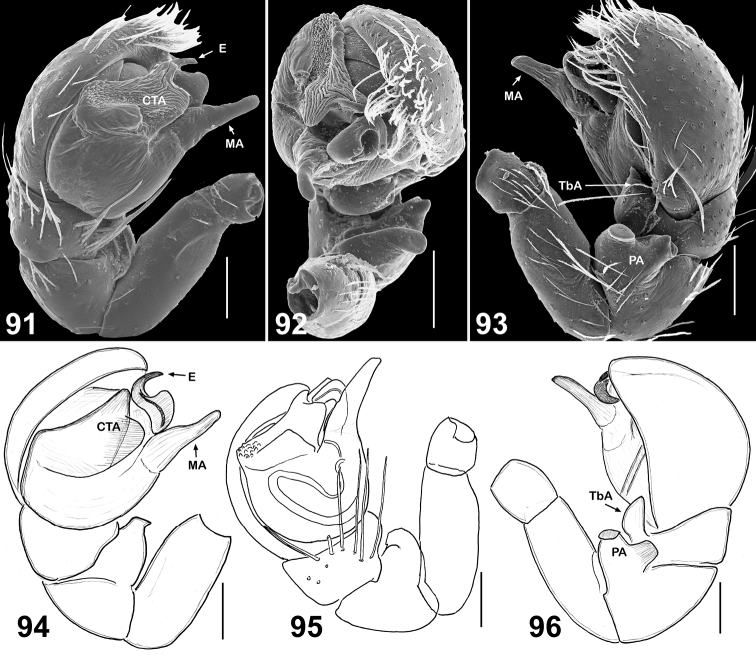
Copulatory organs of *Eupoa pulchella* sp. n. (**91–93**, the holotype; **94–96**, ♂ paratype). **91, 94–95** male palp, median view **92** ditto, apical view **93, 96** ditto, retrolateral view. Abbreviations as explained in ‘Material and methods’. Scale bars: 0.1 mm.

Female unknown.

#### Eupoa schwendingeri sp. n.

http://zoobank.org/58EF7EFD-7F8C-42FF-9719-3DA2BC5C001B

http://species-id.net/wiki/Eupoa_schwendingeri

Figs 89–90 , 97–105

##### Type.

Holotype 1♂ (MHNG) from northern Thailand, Chiang Mai Prov. and Distr., Doi Suthep-Pui National Park, Doi (=Mount) Suthep, 1180 m a.s.l., 1-30.03.1987, P. J. Schwendinger.

Paratypes: THAILAND: 2♀ (MHNG), together with the holotype; 1♂ (MHNG), northern Thailand, Chiang Mai Prov. and Distr., Doi Suthep-Pui National Park, near Pin Pak Pai Waterfall, 1155 m a.s.l., pitfall traps 10.01-11.02.1997, P. Dankittipakul.

##### Etymology.

The species is named in honour of our colleague, the well-known arachnologist, Dr Peter Schwendinger (Geneva, Switzerland), who collected the type series and who made the majority of the *Eupoa* specimens treated in the present work available to us.

##### Diagnosis.

**Figures 97–105.**
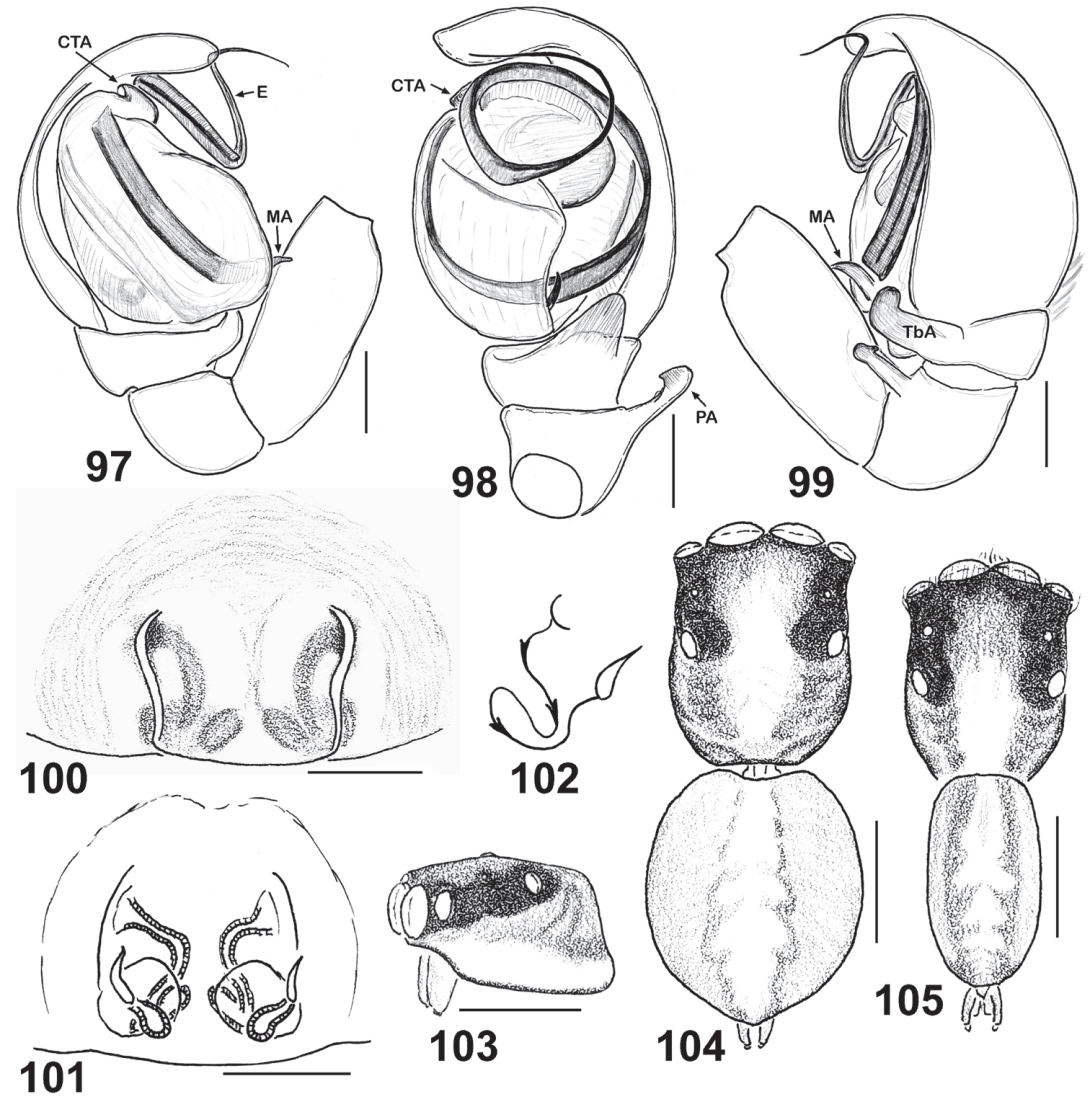
Copulatory organs and somatic characters of *Eupoa schwendingeri* sp. n. (♂ and ♀ paratypes). **97** male palp, median view **98** ditto, ventral view **99** ditto, retrolateral view **100** epigyne, ventral view **101** vulva, dorsal view **102** diagrammatic course of insemination ducts **103** female carapace, lateral view **104** female body, dorsal view **105** male body, dorsal view. Abbreviations as explained in ‘Material and methods’. Scale bars: 0.1 mm (**97–101**), 0.25 mm (**103–105**).

The male palp of *Eupoa schwendingeri* sp. n. differs from those of all *Eupoa* species known to us in having the rather small and inconspicous median and compound terminal apophyses (Figs 97–99). The female of *Eupoa schwendingeri* sp. n. has the unique epigyne shaped as a square plate, with parallel sides (Fig. 100) and the entrances of insemination ducts directed to each other (Fig. 101). Besides, both sexes of this species have a very characteristic body colour pattern consisting of two longitudinal subparallel grey stripes on dorsum (Figs 89, 104–105).

##### Distribution.

The only locality in Thailand: Doi Suthep-Pui National Park.

##### Comments.

The ♂ paratype was lost after having been photographed, as the observation glass was incidentally dropped down to the floor. Thus, the ♂ paratype is represented by the male palp only (Figs 97–99) that is retained in the MHNG.

##### Description.

MALE (the holotype). *Measurements*. Carapace 0.90 long, 0.60 wide and 0.45 high at PLE. Ocular area 0.57 long, 0.73 wide anteriorly and 0.64 wide posteriorly. Diameter of AME 0.23. Clypeus height 0.04, chelicera length 0.30. Abdomen 0.88 long, 0.53 wide. Length of leg segments: I: 0.50 + 0.23 + 0.33 + 0.29 + 0.21; II: 0.40 + 0.16 + 0.23 + 0.26 + 0.20; III: 0.37 + 0.16 + 0.24 + 0.27 + 0.21; leg IV is missing. *Leg spination*. Legs I and II: no spines. Leg III: Mt pr 0-1-0. Leg IV is missing. *Coloration* (Figs 89–90, 104). Carapace yellow, with two wide longitudinal light brown bands. Eye field dark grey, blackened around eyes. Clypeus naked, yellowish brownish. Sternum, labium, maxillae and chelicerae light yellow. Abdomen yellow, with two longitudinal light grey stripes on dorsum. Book-lung covers light yellow. Spinnerets: anterior pair dark grey, posterior pair yellow. Leg I yellow, but femur (its distal part), patella, tibia and metatarsus with black pro-lateral longitudinal stripe, tibia and metatarsus also with light grey retro-lateral longitudinal stripe. Leg II yellow, but tibia ventrally with light grey longitudinal stripe. Legs III and IV yellow. Palps yellow, with brownish cymbia. Palpal structure as in Figs 97–99: patellar apophysis short, fingerlike; tibial apophysis thick and obtuse, directed ventrad with its tip bend dorsad; tegulum developed on its prolateral side only; tegular apophysis poorly developed, looking like an anterior ridge of tegulum; median apophysis spine-shaped, situated at the proximal end of tegulum; compound terminal apophysis poorly developed, looking like a hook-shaped process at the basal part of embolus; embolus coiled, making two revolutions.

FEMALE. *Measurements*. Carapace 0.98 long, 0.76 wide and 0.55 high at PLE. Ocular area 0.63 long, 0.79 wide anteriorly and 0.71 wide posteriorly. Diameter of AME 0.24. Clypeus height 0.07, chelicera length 0.29. Abdomen 0.90 long, 0.68 wide. Length of leg segments: I: 0.54 + 0.20 + 0.35 + 0.34 + 0.21; II: 0.50 + 0.20 + 0.26 + 0.29 + 0.21; III: 0.44 + 0.16 + 0.29 + 0.33 + 0.20; IV: 0.73 + 0.26 + 0.54 + 0.43 + 0.26. *Leg spination*. Leg I: Tb v 2-2-2ap; Mt v 2-2-2ap. Leg II: Tb pr 0-1, v 1-1; Mt v 2-2-2ap. Leg III: Tb pr and rt 0-1-0; Mt pr and rt 1-0. Leg IV: Tb pr and rt 0-1-0; Mt pr and rt 1-1. *Coloration* as in the male (Fig. 105), but palps and both pairs of spinnerets entirely yellow. Epigyne and vulva as in Figs 100–102: epigyne as square-shaped plate, with parallel sides; insemination ducts at their entrances directed to each other and then run posteriorly; receptacles small and pear-shaped.

#### Eupoa thailandica sp. n.

http://zoobank.org/6E26B6C3-1CE6-43E4-8C17-58C4F7CCD61F

http://species-id.net/wiki/Eupoa_thailandica

Figs 85–86 , 106 –118

##### Type.

Holotype 1♂ (MHNG) from Thailand, Trat Prov., Ko Chang, west side (12°03'N, 102°18'E), WINKLER-extraction in secondary forest with primary spots (AS-T-5), 50-200 m a.s.l., 3-23.12.1999, A. Schultz.

Paratypes: THAILAND: 2♂1♀ (MHNG, one male without abdomen and palp), together with the holotype.

##### Etymology.

The species epithet originates from the country of origin of the type series.

##### Diagnosis.

By having the massive compound terminal apophysis with a deep longitudinal groove on its apical edge (Fig. 109, 115), the male of *Eupoa thailandica* sp. n. is most similar to that of *Eupoa pappi* sp. n. (Fig. 67–68), from which it differs in having the smaller and more closed apical cavity of tegulum and the shape of tegular and compound terminal apophyses. The hook-shaped embolic tip of *Eupoa thailandica* sp. n. (Fig. 110) is unique among all the *Eupoa* species known to us. The female of *Eupoa thailandica* sp. n. has a characteristic, mushroom-shaped central epigynal plate (Fig. 117) and the distinct spermathecae (Fig. 118). Besides, both sexes of *Eupoa thailandica* sp. n. have a very characteristic colour pattern on the dorsum containing a large transverse brown spot at its rear end (Figs 85–86); of the *Eupoa* species known to us, only the female of *Eupoa yunnanensis* has got a similar colour pattern (Fig. 121).

**Figures 106–110.**
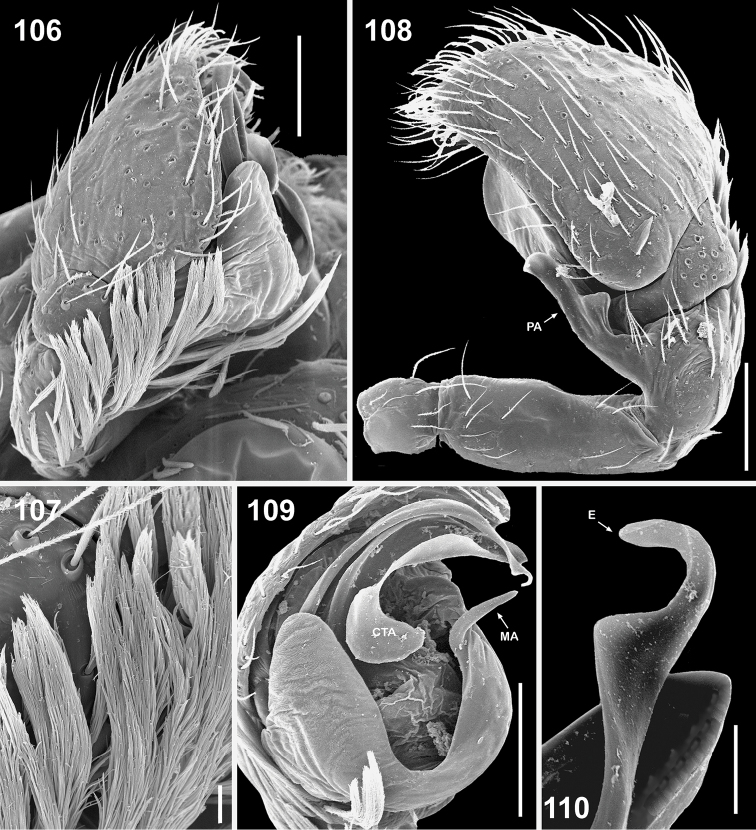
Copulatory organs of *Eupoa thailandica* sp. n. (♂ paratype). **106** male palp, median view **107** bunches of white hairs at the base of cymbium, median view **108** male palp, retrolateral view **109** ditto, ventral view **110** embolic tip, apical view. Abbreviations as explained in ‘Material and methods’. Scale bars: 10 μm (**107, 110**), 0.1 mm (**106, 108–109**).

**Figures 111–118.**
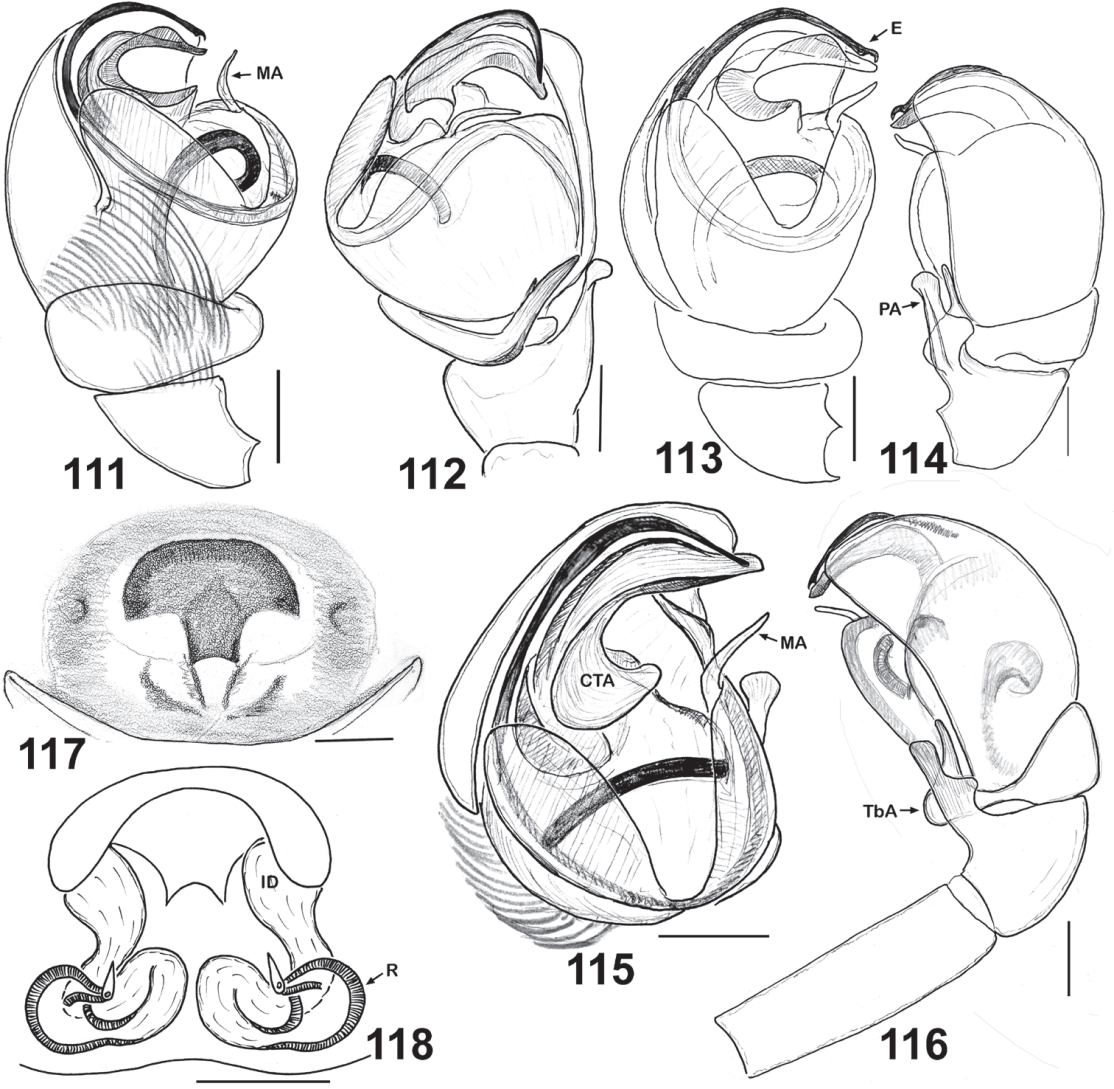
Copulatory organs of *Eupoa thailandica* sp. n. (♂ and ♀ paratypes). **111, 113** male palp, median view **112** ditto, ventral view **114** male palp, dorsal view **115** ditto, ventral view **116** ditto, retrolateral view **117** epigyne, ventral view **118** vulva, dorsa view. Abbreviations as explained in ‘Material and methods’. Scale bars: 0.1 mm.

**Figures 119–122.**
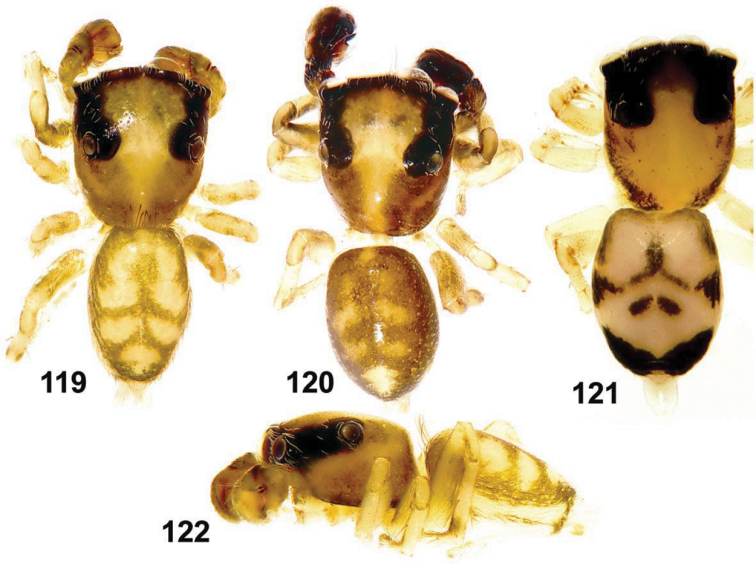
General appearance of *Eupoa yunnanensis* from Laos. **119, 120** male body, dorsal view **121** female body, dorsal view **122** male body, lateral view.

##### Distribution.

The type locality only.

##### Description.

MALE (the holotype). *Measurements*. Carapace 0.87 long, 0.73 wide and 0.54 high at PLE. Ocular area 0.56 long, 0.77 wide anteriorly and 0.66 wide posteriorly. Diameter of AME 0.24. Clypeus height 0.13, chelicera length 0.24. Abdomen 0.67 long, 0.50 wide. Length of leg segments: I: 0.50 + 0.21 + 0.33 + 0.30 + 0.21; II: 0.39 + 0.16 + 0.31 + 0.27 + 0.20; III: 0.39 + 0.17 + 0.26 + 0.27 + 0.18; IV: 0.67 + 0.26 + 0.47 + 0.36 + 0.23. *Leg spination*. Leg I: Tb v 2-2-2ap; Mt v 2-2-2ap. Leg II: Tb v 1-0; Mt v 1-1. Leg III: Tb pr 0-1-0. Leg IV: Tb pr 0-1-0; Mt pr 1ap. *Coloration* (Fig. 86). Carapace yellow, with a brownish marginal stripe at rear end and with brownish eye filed. Blackened around eyes. Clypeus naked, yellow. Sternum, maxillae, labium and chelicerae pale yellow. Abdomen pale yellow, but dorsum with a colour pattern consisting of two brown semi-rings in its fore-half and a transverse brown spot at the rear end (just in front of the spinnerets). Book-lung covers and spinnerets pale yellow. All legs and palps yellow. Palpal structure as in Figs 106–116: femur rather long and thin, almost equal to the length of cymbium; patellar apophysis medium-sized, fingerlike, with a dorso-basal bulge, reaching almost a third of the cymbial length; tibia with set of long hairs prolaterally, tibial apophysis hook-shaped, directed ventrad with its sharp tip bend upward and directed anteriorly; cymbium with a median, basal bunch of thick white hairs; tegulum well-developed; tegular apophysis not developed; median apophysis thin, directed ventrad and situated in the central part of tegulum; compound terminal apophysis massive, as a large, thick hook having a longitudinal groove on its dorsal edge; embolus coiled, making one and a half revolutions, its tip hook-shaped.

FEMALE. *Measurements*. Carapace 0.96 long, 0.77 wide and 0.54 high at PLE. Ocular area 0.59 long, 0.81 wide anteriorly and 0.69 wide posteriorly. Diameter of AME 0.24. Clypeus height 0.07, chelicera length 0.31. Abdomen 1.04 long, 0.91 wide. Length of leg segments: I: 0.54 + 0.21 + 0.36 + 0.34 + 0.20; II: 0.44 + 0.19 + 0.26 + 0.27 + 0.20; III: 0.41 + 0.17 + 0.30 + 0.31 + 0.21; IV: 0.73 + 0.22 + 0.59 + 0.43 + 0.25. *Leg spination*. Leg I: Tb v 2-2-2ap; Mt v 2-2-2ap. Leg II: Tb pr 0-1, v 1-2; Mt pr 1-1, rt 0-1-0, v 2-0-2ap. Leg III: Tb pr and rt 0-1-0; Mt pr and rt 0-1-0. Leg IV: Tb pr 0-1-0; Mt pr and rt 1-0-1ap. *Coloration* as the male, but the dark brown spot on dorsum is larger (Fig. 85). Epigyne and vulva as in Figs 117–118: central epigynal plate mushroom-shaped, sclerotized and contrastingly darker than the rest of epigyne; wide transparent insemination ducts; round receptacles that are spaced up.

#### Eupoa yunnanensis

Peng & Kim, 1997

http://species-id.net/wiki/Eupoa_yunnanensis

Figs 119 –128

Eupoa yunnanensis Peng & Kim, 1997: 196, figs 3A–C (D♂).
Eupoa yunnanensis : Song et al. 1999: 509, figs 292O, 325J (♂).

##### Material.

Laos: 2♂ (SMFM), Luang Prabang Prov., NE Luang Prabang, Nam Ou, Nong Khiao, Tham Pathok (20°33.082'N, 102°37.925'E'), 373 m a.s.l., outside cave, sieving leaf litter, 17–18.03.2007, P. Jäger & F. Steinmetz; 2♀ (SMFM), Luang Prabang Prov., SE Luang Prabang, Xieng Nguen Distr., Nam Khan, Ban Keng Koung (19°40.963'N, 102°18.442'E), 372 m a.s.l., along stream, sieving leaf litter, 22.03.2007, P. Jäger; 1♀ (SMFM), same locality, disturbed forest, dry stream bed, sieving and WINKLER-extraction, 21-23.02.2008, P. Jäger.

##### Diagnosis.

The male of *Eupoa yunnanensis* has the unique, widest and strongest patellar apophysis (Figs 124, 126) among all the *Eupoa* species known to us. The female has the unique conformation of its epigyne: viz., the singular atrium formed by the posterior chitinous margin and the anterior transverse pocket (Fig. 127) and the S-shaped spermathecae (Fig. 128).

**Figures 123–128.**
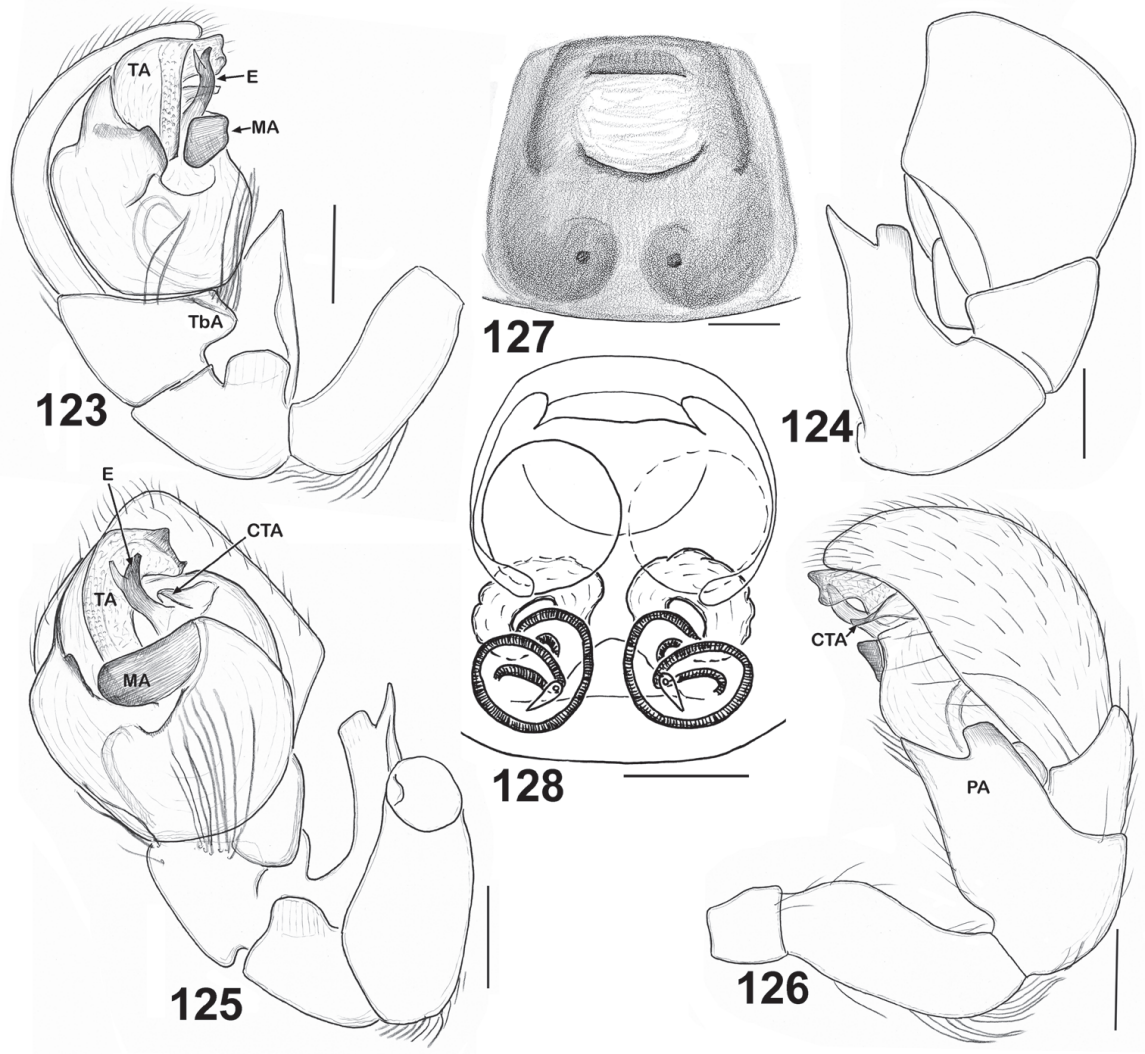
Copulatory organs of *Eupoa yunnanensis* from Laos. **123** male palp, median view **124** ditto, dorsal view **125** ditto, ventral view **126** ditto, retrolateral view **127** epigyne, verntral view **128** ditto, dorsal view. Abbreviations as explained in ‘Material and Methods’. Scale bars: 0.1 mm.

##### Distribution.

China (Yunnan) (Peng and Kim 1997) and northern Laos (Luang Prabang province) (present data).

##### Description.

MALE. *Measurements*. Carapace 0.96 long, 0.79 wide and 0.50 high at PLE. Ocular area 0.60 long, 0.83 wide anteriorly and 0.75 wide posteriorly. Diameter of AME 0.29. Clypeus height 0.06, chelicera length 0.21. Abdomen 0.83 long, 0.50 wide. Length of leg segments: I: 0.49 + 0.19 + 0.30 + 0.31 + 0.21; II: 0.43 + 0.20 + 0.23 + 0.27 + 0.21; III: 0.43 + 0.19 + 0.24 + 0.31 + 0.26; IV: 0.64 + 0.24 + 0.47 + 0.39 + 0.29. *Leg spination*. Leg I: Mt v 2-2-2ap. Leg II: no spines. Leg III: Tb pr and rt 0-1-0. Leg IV: Tb pr and rt 0-1-0; Mt d, pr and rt 1-1ap. *Coloration* (Figs 119–120, 122). Carapace brownish, with a wide longitudinal median yellow stripe, the area between PLEs also yellow; blackened around eyes. Clypeus naked, brownish yellow. Sternum, labium, maxillae and chelicerae yellow. Abdomen: dorsum and sides dark grey, with two longitudinal rows of yellow spots on dorsum; dorsum with shining scutum; venter yellow. Book-lung covers yellow. Spinnerets: anterior pair dark grey, posterior pair yellow. All legs yellow, but femora I and patellae I grey on their sides, tibiae I ventrally grey and patellae II-IV grey on their sides. Palps brownish. Palpal structure as in Figs 123–126: patellar apophysis thick, massive and split at its tip, reaching almost a half of the cymbial length; tibial apophysis short and wide, poorly-developed; tegulum well-developed; tegular apophysis wide and strong, looking like a median extension; median apophysis thick and visibly sclerotized, situated in the central part of tegulum; compound terminal apophysis short, situated at the basis of embolus; embolus fingerlike, with a short hook-shaped process on its tip.

FEMALE. *Measurements*. Carapace 1.13 long, 0.86 wide and 0.60 high at PLE. Ocular area 0.59 long, 0.89 wide anteriorly and 0.76 wide posteriorly. Diameter of AME 0.29. Clypeus height 0.06, chelicera length 0.20. Abdomen 1.10 long, 0.85 wide. Length of leg segments: I: 0.65 + 0.28 + 0.48 + 0.40 + 0.25; II: 0.53 + 0.23 + 0.35 + 0.35 + 0.23; III: 0.51 + 0.23 + 0.38 + 0.40 + 0.25; IV: 0.90 + 0.33 + 0.73 + 0.50 + 0.30. *Leg spination*. Leg I: Tb v 2-2-2ap; Mt v 2-2-2ap. Leg II: Tb pr and rt 0-1-0, v 1-2; Mt v 2-2-2ap. Leg III: Tb pr and rt 0-1-0; Mt pr and rt 1-0. Leg IV: Tb pr and rt 0-1-0; Mt pr 1-0-2ap, rt 1-0-1ap. *Coloration* (Fig. 121). Carapace yellow, with brownish margins and brownish eye field; blackened around eyes. Clypeus naked and yellow. Sternum, labium, maxillae and chelicerae yellow. Abdomen: dorsum yellow, with two dark brown pattern consisting of Λ-shaped figure and two round merging spots at its rear; venter yellow. Book-lung and spinnerets yellow. All legs and palps yellow. Epigyne and vulva as in Figs 127–128: central atrium present, it is formed by the posterior chitinous margin and the anterior transverse pocket; relatively short, transparent insemination ducts make a C-shaped loop; there are also visible large transparent sacs that seem to be disconnected from the insemination ducts (in the studied vulva only one sac is left); sclerotized receptacles bean-shaped.

## Supplementary Material

XML Treatment for Eupoa

XML Treatment for Eupoa daklak

XML Treatment for Eupoa lehtineni

XML Treatment for Eupoa lobli

XML Treatment for Eupoa pappi

XML Treatment for Eupoa prima

XML Treatment for Eupoa pulchella

XML Treatment for Eupoa schwendingeri

XML Treatment for Eupoa thailandica

XML Treatment for Eupoa yunnanensis
